# Micro-CT Technique Is Well Suited for Documentation of Remodeling Processes in Murine Carotid Arteries

**DOI:** 10.1371/journal.pone.0130374

**Published:** 2015-06-18

**Authors:** Christoph Schürmann, Felix Gremse, Hanjoong Jo, Fabian Kiessling, Ralf P. Brandes

**Affiliations:** 1 Institut für Kardiovaskuläre Physiologie, Fachbereich Medizin der Goethe-Universität, Theodor-Stern-Kai 7, Frankfurt am Main, Germany; 2 Experimental Molecular Imaging, University Clinic Aachen, RWTH Aachen University, Aachen, Germany; 3 Wallace H. Coulter Department of Biomedical Engineering, Georgia Institute of Technology and Emory University, Atlanta, Georgia, United States of America; 4 German Center for Cardiovascular Research (DZHK), Partner site RheinMain, Frankfurt, Germany; University of Buenos Aires, Faculty of Medicine. Cardiovascular Pathophysiology Institute., ARGENTINA

## Abstract

**Background:**

The pathomechanisms of atherosclerosis and vascular remodelling are under intense research. Only a few in vivo tools to study these processes longitudinally in animal experiments are available. Here, we evaluated the potential of micro-CT technology.

**Methods:**

Lumen areas of the common carotid arteries (CCA) in the ApoE^-/-^ partial carotid artery ligation mouse model were compared between in vivo and ex vivo micro-CT technique and serial histology in a total of 28 animals. AuroVist-15 nm nanoparticles were used as in vivo blood pool contrast agent in a Skyscan 1176 micro-CT at resolution of 18 μmeter voxel size and a mean x-ray dose of 0.5 Gy. For ex vivo imaging, animals were perfused with MicroFil and imaged at 9 μmeter voxel size. Lumen area was evaluated at postoperative days 7, 14, and 28 first by micro-CT followed by histology.

**Results:**

In vivo micro-CT and histology revealed lumen loss starting at day 14. The lumen profile highly correlated (r = 0.79, P<0.0001) between this two methods but absolute lumen values obtained by histology were lower than those obtained by micro-CT. Comparison of in vivo and ex vivo micro-CT imaging revealed excellent correlation (r = 0.83, P<0.01). Post mortem micro-CT yielded a higher resolution than in vivo micro-CT but there was no statistical difference of lumen measurements in the partial carotid artery ligation model.

**Conclusion:**

These data demonstrate that in vivo micro-CT is a feasible and accurate technique with low animal stress to image remodeling processes in the murine carotid artery.

## Introduction

Murine models are a preferred tool to study atherosclerosis because disease development is rather fast, breeding capacity is high by fairly low costs, and thousands of knockout mice are available to foster mechanistic research.

A well characterized model of accelerated atherogenesis development is the unilateral partial ligation model of the left common carotid artery (LCCA) in apolipoprotein E knockout (ApoE^-/-^) mice [1,2]. The model is well-suited to study the biophysical and molecular mechanisms of atherosclerosis but also to identify anti-atherosclerotic agents [3–5]. The latter purpose necessitates studying disease development longitudinally and non-invasively, in a fast and affordable manner. Due to the small animal size, histology is currently the gold standard of atherosclerosis quantification of the carotid artery [6] of the mouse but serial sectioning is time consuming and does not allow for longitudinal data acquisition. Moreover, sample preparation can introduce artefacts by tissue shrinking and distortions during sectioning. Thus, there is a need for accurate in vivo vascular imaging technique to study atherosclerosis development in mice.

Several non-invasive in vivo imaging techniques for rodents have been developed including contrast-enhanced magnetic resonance imaging [7], near-infrared fluorescence (NIRF) imaging [4], positron emission tomography or micro-CT [8]. The primary advantage of the latter technique is high resolution at fairly low cost.

CT imaging of the vascular lumen in general requires contrast agents as the native absorbance difference of blood and tissue does not allow distinguishing these compartments by X-ray technology. The long scanning duration of micro-CT devices and the fast metabolism of mice, however, limit the suitability of low-molecular weight clinical contrast agents for this purpose. Therefore, several blood pool contrast agents with high-molecular weight which are retained in the vascular system of the mouse have been developed. In the present study, AuroVist, which is based on gold nanoparticles and shows favorable toxicity and slow clearance, was used [9]. The compound has greater specific X-ray absorbance than equivalent dose of iodinated contrast agents and therefore yields a higher contrast. Other agents to mouse imaging have been developed like eXIA 160 XL (Binitio Biomedical, Inc, Ottawa, Ontario, Canada) and should in principle yield similar image quality.

For ex vivo imaging, X-ray dose and excretion is irrelevant, and numerous contrasting techniques are available. Interesting approaches are the combination of latex beads with barium to selectively image the arterial or venous vascular bed although most studies rely on the polymerizing contrast agent MicroFil [10,11], which combines the advantage of high x-ray absorbance with perfect vascular retention and formation of stable vascular cast preparations.

Histological analysis of serial sections is the gold standard for atherosclerosis studies [6]. The method allows not only the determination of the vascular lumen but also the analysis of the vascular wall composition. Although in vivo micro-CT has been used in experimental models of atherosclerosis, the technique has not been carefully validated against histology. The few studies performed, employed ex vivo imaging but the high resolution post mortem micro-CT scans have not been correlated with in vivo measurements for the CCA lumen area of mice.

We hypothesized that in vivo micro-CT imaging is a suitable technique to study vascular luminal remodelling in the CCA of mice and studied this in the particular carotid ligation model of ApoE^-/-^ mice.

## Materials and Methods

### Animal experiments

ApoE^-/-^ mice were purchased from Taconis M&B A/S (Ry, Denmark, strain B6.129P2-Apoe^tm1Unc^ N6) and bred at the local facility under standard conditions with 12/12 hour dark / light cycle and free access to chow and water. Western-type (42% of total calories from fat; 0.15% cholesterol) diet was purchased from Harlan Teklad Germany (Harlan Winkelmann, Borchen, Germany).

All animal experiments were performed in accordance with the National Institutes of Health Guidelines on the Use of Laboratory Animals. The University Animal Care Committee and the Federal Authorities for Animal Research (Darmstadt, Germany) approved the study protocol.

**Fig 1:**
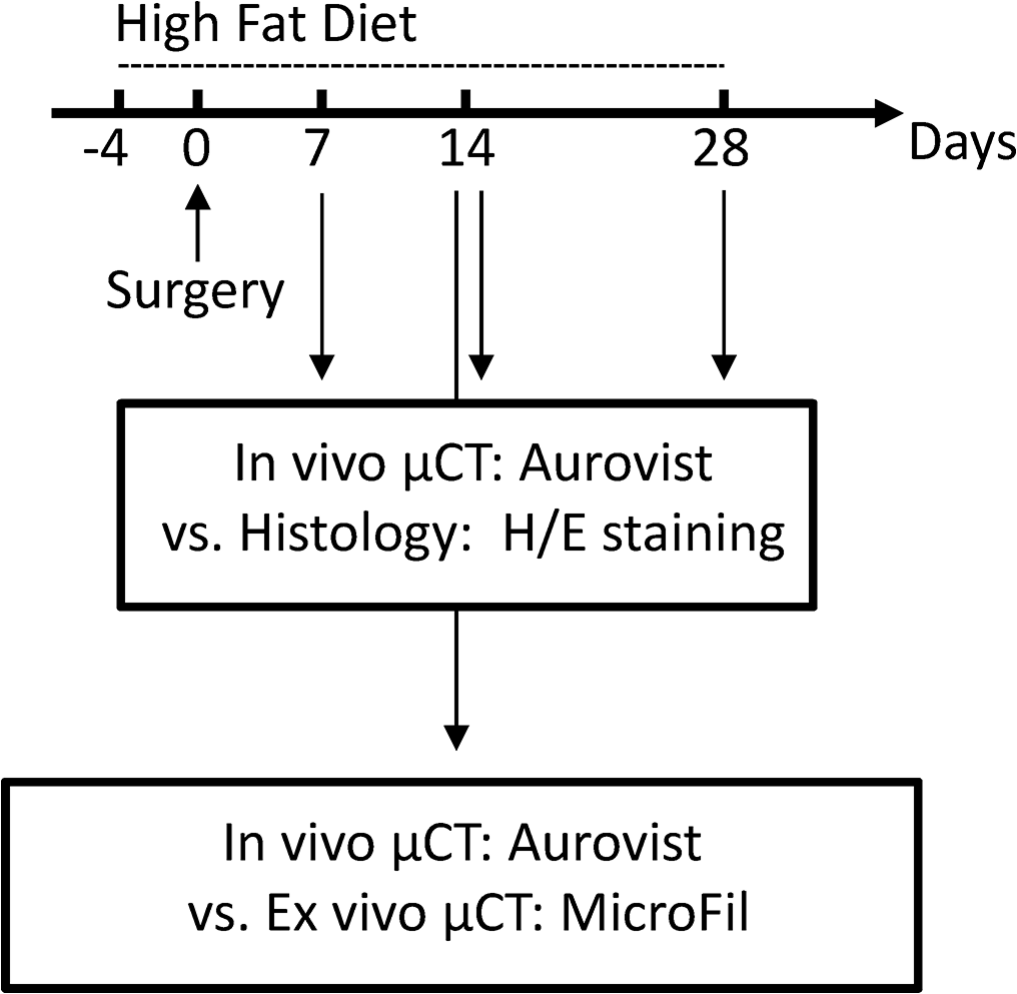
Experimental design. Partial ligation of the LCCA in ApoE^-/-^ mice at day 0. Timeline of high fat diet (HFD) treatment and overview showing analysis methods. Comparison of in vivo micro-CT and histology was performed by in vivo micro-CT scan and subsequent histological examination (d7, n = 4, d14, n = 10, d28, n = 9). Comparison of in vivo micro-CT and ex vivo micro-CT was performed by in vivo micro-CT scan and subsequent perfusion with the post mortem contrast agent MicroFil (n = 5); H/E, hematoxylin/eosin.

Animals were started on Western-type diet at the age of 10 weeks (**Fig 1**). Neo-intima formation and accelerated atherosclerosis was induced four days later by partial left common carotid artery ligation as described previously [1]. The model is superior to the total ligation model as the remaining blood flow through the superior thyroid artery prevents vascular clotting.

Animals were divided into four groups (n = 28). In three groups in vivo micro-CT was correlated to histology whereas in the fourth group ex vivo and in vivo micro-CT were correlated. In order to compare CCA lumen area between in vivo micro-CT and histology in vivo micro-CT scans were performed at postoperative days 7 (n = 4), 14 (n = 10) or 28 (n = 9), and subsequently, at each time point, mice were sacrificed and prepared for histology. Moreover, five animals were used to compare CCA lumen area between in vivo and ex vivo post mortem micro-CT at postoperative day 14.

### In vivo micro-CT

Animals were anesthetized with isoflurane (1%) and AuroVist (100 microliter—Nanoparticles 15 nm, Nanoprobes, NY, USA) was injected through the tail vein. The mice were positioned supine in the sample holder of the micro-CT (Skyscan 1176, Bruker micro-CT, Kontich, Belgium). To reduce motion artefacts, the neck of the mouse was fixed by polystyrene foam. Imaging was performed without gating, because this increased the scanning duration and did not substantially improve the image quality. Scan parameters were optimised to reduce scanning time and radiation dose. For the present study a radiation dose of 470 mGy in a 13 min. scan was applied with the following settings: 50 kV source voltage, 1 mm aluminium filter, 250 μA source current, exposure time 750 ms, 18 μm isotopic resolution, 1 projection image per 0.5° gentry rotation step, rotation range 360° and a field of view (FOV) covering the neck region.

### Mouse perfusion

For ex vivo imaging and histology, animals were sacrificed directly after the in vivo micro-CT scan and perfused with vasodilation buffer (5 min, PBS, papaverine 4 mg/L, adenosine 1 g/L, Sigma-Aldrich, Taufkirchen, Germany) followed by intravascular paraformaldehyde fixation (4% PFA, 15 min). For this, a 24G x ¾ catheter was inserted into the abdominal aorta and connected to PE tubing for retrograde perfusion.

### Ex vivo micro-CT

Following intravascular PFA fixation, the vascular system of the mouse was perfused with the radiopaque contrast agent MicroFil (Flow Tech, Carver, MA, USA) with a pressure of 100–120 mmHg until the silicon polymer cured. Mice were stored overnight in 4% PFA at 4°C and subsequently scanned in the same micro-CT as used for in vivo imaging (Skyscan 1176, Bruker micro-CT, Kontich, Belgium) with the following settings: 50 kV, 0.5 mm aluminium filter, 500 μA source current, exposure time 875 ms, 9 μm isotopic resolution, 3 projection images per 0.3° rotation step, rotation range 360° and a field of view (FOV) covering the entire carotid artery.

### Three dimensional reconstruction and image analysis

**Fig 2:**
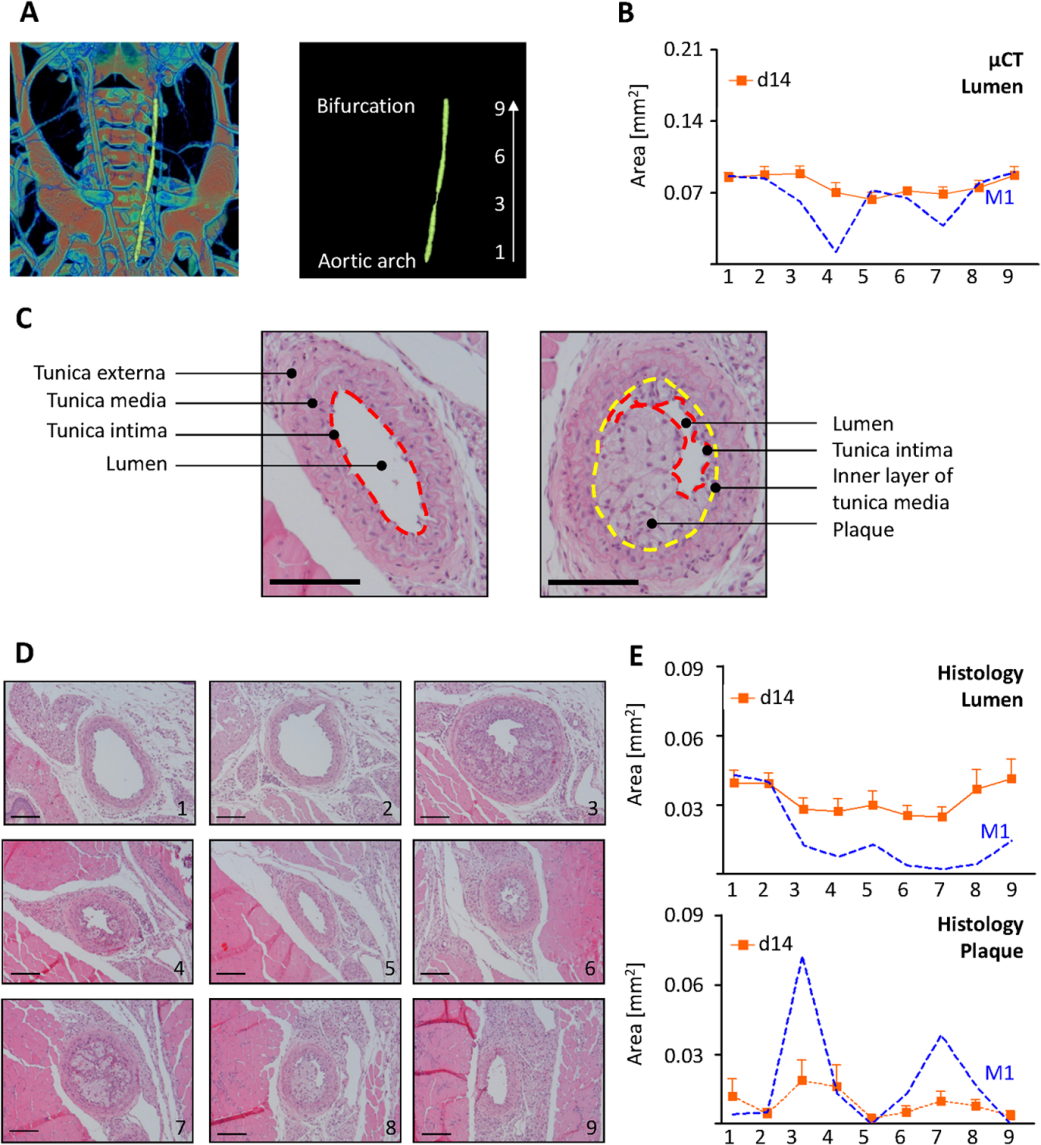
Lumen profile evaluation by in vivo micro-CT and histology (A) Volume rendering of a micro-CT angiography of a ApoE^-/-^ mouse infused with AuroVist nanoparticles at postoperative day 14 after partial LCCA (left common carotid artery) ligation. A virtual elastic sphere (yellow) is fitted through the segmented LCCA lumen (left panel) from the aortic arch to the bifurcation. The LCCA was segmented into nine equidistant parts (right panel). (B) Side dependent vascular lumen profile of the LCCA from the aortic arch (1) till the bifurcation (9) Shown are the averages of all mice (n = 10) in orange and mouse M1, corresponding to (A), in blue. (C) H/E stainings of LCCA at postoperative day 14 after partial ligation of the LCCA. Lumen, tunica intima (red dashed line), tunica media, tunica externa, inner layer of tunica media (yellow dashed line) and plaque are clearly seen. (D) H/E stainings of LCCA (M1) at postoperative day 14 near the aortic arch (1), the bifurcation (9) and each 500 μm in between. (E) Histological evaluation of LCCA lumen (upper panel) and plaque area (lower panel) from the aortic arch (1) to the bifurcation (9) (n = 10). Squares show the mean ± SEM of all mice. Dashed blue line shows one individual mouse (M1). Scale bars in (C) and (D) represent 100 μm.

Volumetric data was reconstructed with the NRecon/InstaRecon CBR Server–Premium software (Skyscan, Kontich, Belgium/ InstaRecon, Champaign, Illinois, USA). Image analysis, segmentation of micro-CT data and quantification of carotid artery lumen area were performed with the Imalytics Preclinical Software (Gremse-IT, Aachen, Germany). Discrimination of contrast-enhanced blood and soft tissue was achieved by applying a threshold defined as the mean of blood and muscle intensity [12]. The lumen areas of the LCCA and RCCA were measured with the virtual elastic sphere tool [12]. The start point was set in the CCA shortly behind the aorta and the end point shortly before the bifurcation (**Fig 2A**). The luminal diameters along the CCA were obtained though the elastic sphere tool, grouped, and averaged over nine equidistant parts (**Fig 2B**).Vessel area was calculated from the CCA diameters assuming circular cross-section. In order to compare lumen area measurements with histology data and to normalize varying CCA lengths between animals, the lengths of the carotid arteries was normalized and partitioned into nine equidistant parts. The vascular profiles are created for lumen area and for plaque area.

### Histology

After intravascular PFA-perfusion, the heart and the soft tissue of the neck were removed, fixed in 4% PFA overnight and subsequently embedded in paraffin. Sections of 3 μm were cut every 500 μm from the aortic arch to the bifurcation. The lumen of the carotid artery was determined by planimetry in hematoxylin and eosin H/E-stained sections with the aid of the NIH-ImageJ software.

In general, determination of the vascular lumen area was performed by planimetry of the lumen surrounded by tunica intima. In few cases vessels became compressed during paraffin embedding (**Fig 2C, left panel**). Therefore the luminal diameter was always obtained from the luminal perimeter, assuming a circular shape. Luminal areas of plaque-loaded vessels were determined by measuring the perimeter of the inner layer of the tunica media (**Fig 2C, right panel**). Next, the area of the plaque was subtracted from the calculated area of the circle to estimate the lumen area.

### Statistical analysis

Data are shown as means ± SEM. Correlations between micro-CT data and histological analysis were performed by linear regression. Statistical analyses were carried out in Graph Pad Prism 5. Statistical analysis of time dependant lumen loss was carried out with means of Section 1–9 by ANOVA with Bonferroni correction. Comparison of lumen or plaque area along the carotid artery was performed by student´s T-test. Correlations between in vivo and ex vivo micro-CT data were performed by linear regression. Values of p<0.05 were considered statistically significant.

## Results

### Micro-CT and histology reveal similar lumen profiles

The lumen profile of the CCAs in ApoE^-/-^ mice at postoperative day 14 was analyzed by in vivo micro-CT imaging and histology (**Fig 2B and 2C**). Histological sections were obtained every 500 μm starting from the aortic arch to the bifurcation, which resulted in nine sections for each mouse. In vivo micro-CT was performed with a nominal resolution of 18 μm and thereby providing lumen area measurements at this scale of accuracy.

As shown for all animals for day 14 and as exemplified for mouse one (**M1, Fig 2**), in vivo micro-CT and histology both revealed a lumen area profile along the LCCA. For the individual mouse (M1) it is apparent that lumen areas in section 4 and 7 by micro-CT (**Fig 2B**) and4, 6, 7, and 8 by histology (**Fig 2E, upper panel**) were clearly reduced as compared to other sections. Moreover, histology revealed an increase plaque area in section 3, 4, 6, 7 and 8 (**Fig 2E lower panel**). Nevertheless, statistical analysis revealed only minor regional differences in lumen and plaque area at day 14 (S1 Table).

### Vascular lumen profile correlates well between micro-CT and histology

**Fig 3:**
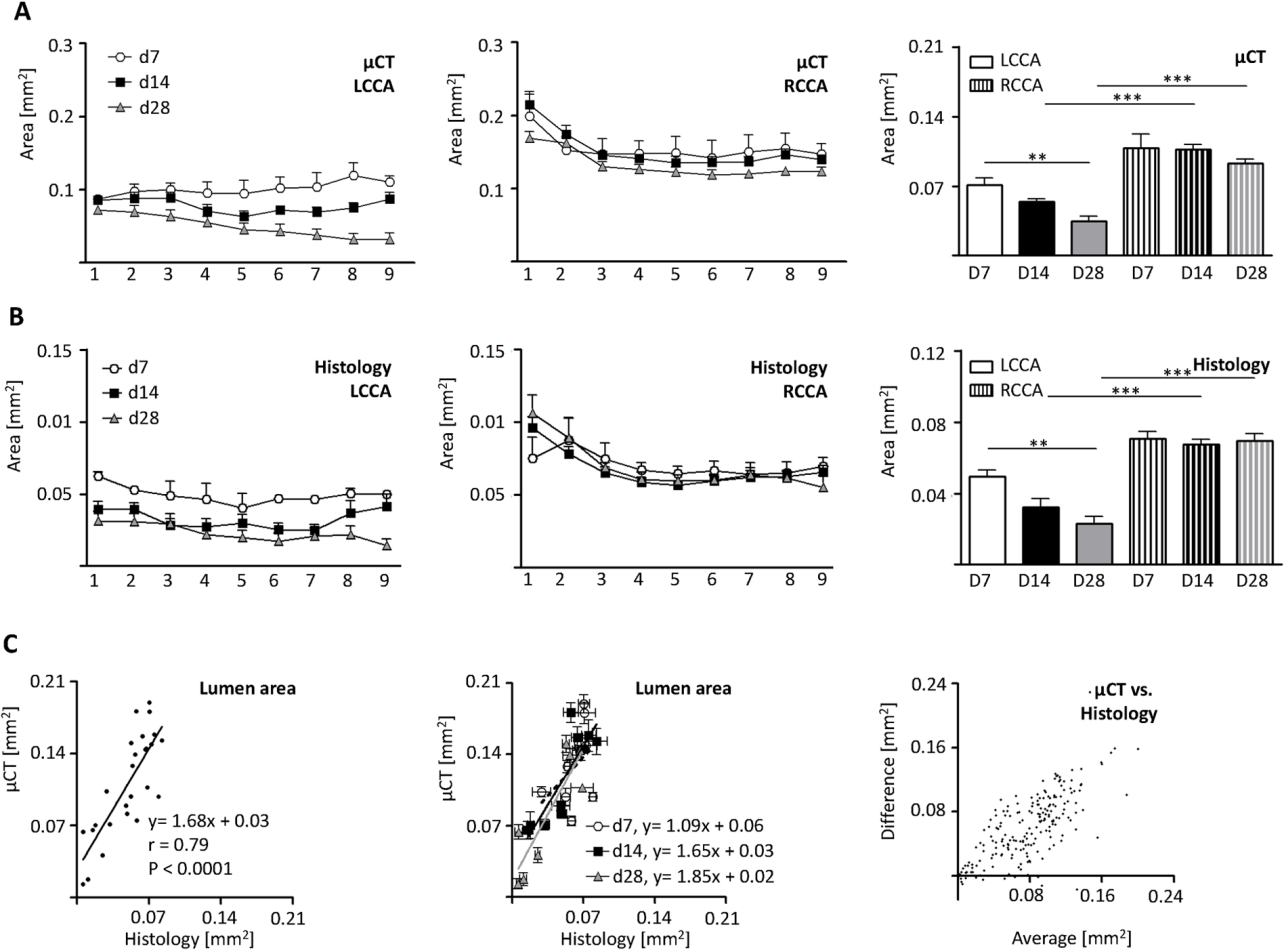
Comparison of in vivo micro-CT and histology vascular lumen profile. LCCA and RCCA lumen profiles evaluated by in vivo micro-CT and histology at postoperative days 7 (n = 4), 14 (n = 10) and 28 (n = 9) after LCCA ligation. (A) In vivo micro-CT evaluation of LCCA (left panel) and RCCA (middle panel) lumen area starting from the aortic arch (1) to the bifurcation (9). Average lumen areas evaluated by micro-CT (right panel). (B) Histological evaluation of LCCA (left panel) and RCCA (middle panel) lumen area profiles. Averages of carotid lumen areas evaluated by histology (right panel). (C) Comparison between mean lumen areas of LCCA and RCCA of each mouse evaluated by in vivo micro-CT and histology. Linear regression and correlation coefficient were calculated (C, left panel). Correlation of lumen areas of LCCA and RCCA of each mouse for each time point (C, middle panel). Bland-Altman plot depicting the degree of agreement between micro-CT and histology (C, right panel). Bars indicate the mean ± SEM. **, p<0.01; ***, p<0.001; LCCA left common carotid artery; RCCA right common carotid artery.

Although partial ligation of the LCCA is a common model of accelerated atherosclerosis, the lumen profile over the whole length of the vessel has not been reported. At post-operative day 7 and 14, micro-CT analysis revealed no differences in lumen area between sections near the aortic arch and the bifurcation (**Fig 3A, left panel**). At a later time point, (day 28) lumen area decreased from proximal to distal. Statistical analysis revealed a negative slope (y = -0.01+0.07), significantly different from that of the earlier time points (P = 0.0001).

The lumen of the RCCA is larger near the bifurcation (section 1 & 2), but remained constant throughout the observation period (**Fig 3A, middle panel**). In contrast, the lumen area of the LCCA significantly decreased over time (**Fig 3A, right panel**) and was reduced compared to the RCCA at postoperative day 14 and 28. Histological analysis of the LCCA and RCCA revealed similar results and significances (**Fig 3B, left and middle panel**).

To assess the correlation between the two techniques, lumen areas from each LCCA and RCCA of all mice were analyzed. The two methods showed a good correlation (r = 0.79, p<0.0001). Linear regression analysis, however, revealed that the lumen by histology was measured approximately 1.7 times smaller than that of the corresponding in vivo micro-CT data (**Fig 3C, left panel**). This effect was preserved when each time point was analyzed separately and also apparent in the Bland-Altman presentation **(Fig 3C, middle and right panel)**.

### In vivo micro-CT and post mortem micro-CT yield similar lumen data

**Fig 4:**
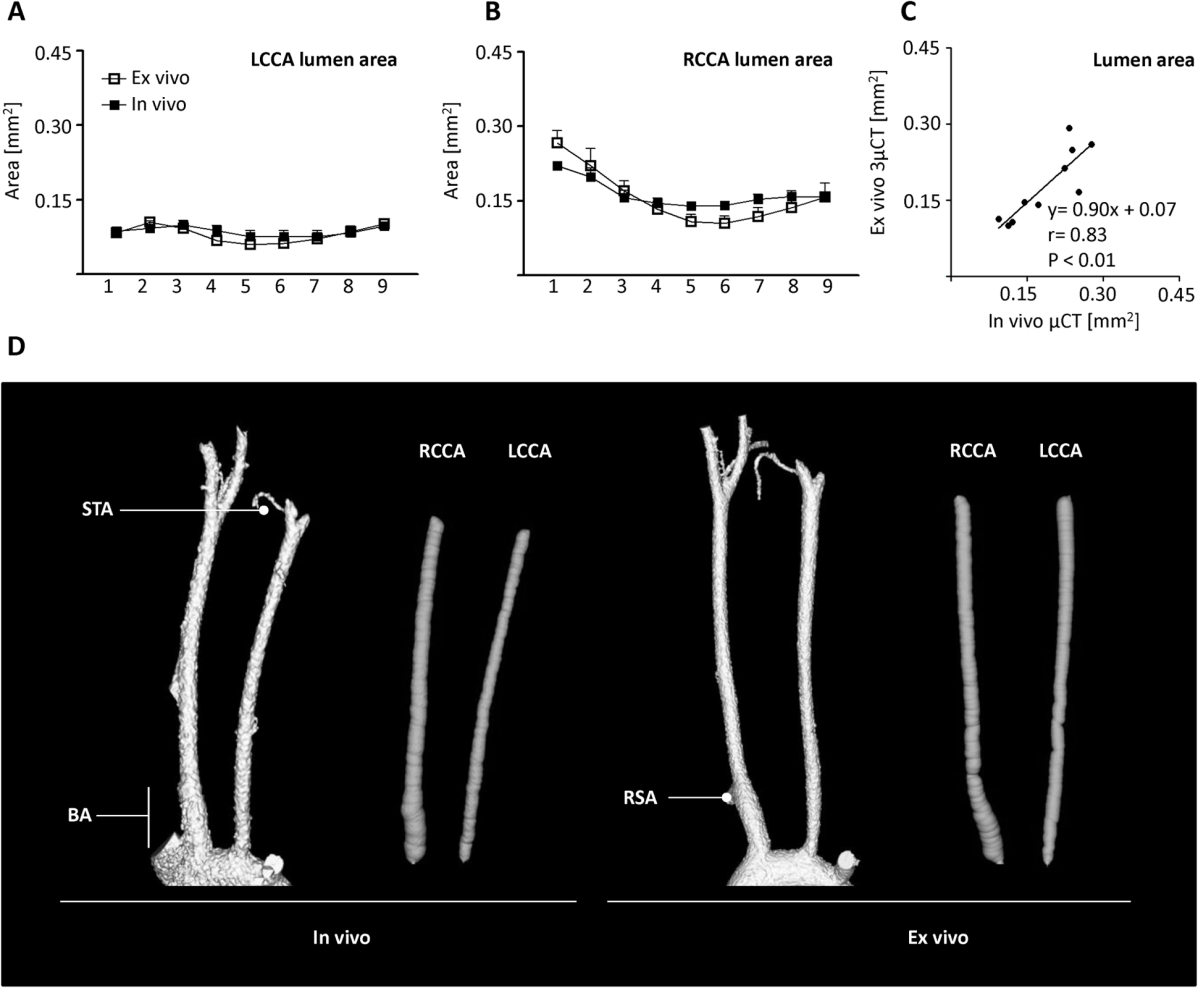
Comparison of in vivo and ex vivo micro-CT. Lumen area profile after partial ligation of the LCCA. (A, B) Lumen profile of the LCCA and RCCA was evaluated by in vivo (AuroVist Nanoparticles) and ex vivo (MicroFil) micro-CT at postoperative day 14 (n = 5). Lumen area was evaluated from the aortic arch (1) to the bifurcation (9). Error bars indicate the mean ± SEM. (C) Correlation between mean lumen area of LCCA and RCCA of each mouse examined by in vivo and ex vivo micro-CT. Linear regression parameters and correlation coefficients were calculated. (D) Volume rendering of the LCCA and RCCA examined by in vivo and post mortem micro-CT with corresponding visualization of the virtual elastic sphere path (grey). BA, brachiocephalic artery; LCCA, left common carotid artery; RCCA right common carotid artery; RSA, right subclavian artery branch; STA, superior thyroid artery.

A well described method to determine vascular lumen area is the post mortem perfusion with the radiopaque silicon compound MicroFil (**Fig 4C**). 14 days after induction of atherogenesis, vascular lumen profile of the same animals was compared between in vivo and post mortem micro-CT (**Fig 4**). The lumen area of the LCCA was similar between both methods (**Fig 4A, left panel**). Interestingly, although not statistically significant, for the RCCA post mortem micro-CT evaluation of the brachiocephalic artery (section no. 1) yielded greater values for ex vivo lumen area than for in vivo measurements (**Fig 4A, right panel**).

The correlation of lumen areas examined by in vivo and post mortem micro-CT was carried out with mean data from each LCCA and RCCA for all 5 mice (**Fig 4B**). The two methods show a good correlation (r = 0.83, p<0.01). Linear regression analysis did not provide evidence for high values in the in vivo micro-CT technique. Thus, although slightly inferior in quality, in vivo imaging yields comparable morphometric data which did not significantly differ from the post mortem micro-CT data.

## Discussion

The unilateral partial carotid ligation model in mice was developed more than ten years ago [13] and underwent intense research [1]. In this study, micro-CT imaging was used to quantify LCCA and RCCA lumen loss during atherosclerotic remodelling processes. Our evaluation demonstrated the suitability of the micro-CT technique to assess the lumen area as a function of time and site along the carotid artery in the partial ligation model in ApoE mice. Interestingly, in vivo and ex vivo micro-CT provided similar measurements, whereas the previous gold standard, histology, resulted in an underestimation of lumen area. Furthermore, serial sectioning is time consuming and does not allow longitudinal data acquisition. The technique is also very laborious, requires a high level of training and is prone to observer bias and preparation artefacts due to sample shrinkage or false positioning for sectioning. Micro-CT, in contrast, is fast and offers the advantage of non-destructive imaging followed by computer-facilitated reconstruction. The resulting 3D image can be freely rotated and analyzed independently of the original imaging orientation. This is of particular importance for the two common carotid arteries (CCA) as these vessels do not run entirely parallel in the neck.

It is known since long that crosslinking and dehydration result in sample shrinkage [14]. Ex vivo micro-CT, however, is confronted with similar problems. Also for this technique the tissue is cross-linked and curing of the polymer also induces some shrinkage of the cast. In contrast, in vivo imaging allows analysis of the vessels in their natural physiological state, i.e. distended by the blood pressure. In a study analyzing aortic arch and CCA with the contrast agent Fenestra VC-131 and Batson´s No.17 casting solution, indeed a 27% smaller volume of the CCA was measured ex vivo [15]. Moreover Kratky et al. [16] demonstrated a shrinking factor of 16–20% for Batson´s No 17. Thus, a significant portion of the difference obtained in those prior studies can be explained by the use of Batson’s No 17, which heavily shrinks. MicroFil in contrast, undergoes much less shrinkage and our approach to keep the perfusion pressure high until the agent had cured completely was sufficient to conserve the in vivo situation.

Nevertheless, the great similarity of the data obtained by ex vivo and in vivo scanning in the present study is surprising as at least theoretically, thresholding at the border of the detection limit is affected by contrast and resolution. For in vivo imaging, an 18 μm resolution was used and the mean diameters of the LCCA and RCCA were 0.328 ± 0.007 mm and 0.454 ± 0.007 mm respectively. Small differences in thresholds will result in under- or overestimation of vessel size. This is important as ex vivo blood intensity (ex vivo: 155.8 ± 1.6 intensity units vs. in vivo: 148.3 ± 2.0 intensity units, p<0.05) is higher and thus threshold determination and image analysis can be more accurate. Our data, however, provide evidence that in 3-dimensional space this consideration is of minor importance. Nevertheless, histology is still the method of choice to analyze atherosclerosis in the aortic root or aortic arch as cardiac and respiratory motions are hard to compensate during in vivo micro-CT. It has to be shown whether ex vivo micro-CT is able to identify atherosclerotic changes in other vessels than the carotid arteries.

It is noteworthy that the present observations cast some doubt on the current histological strategy to quantify the degree of stenosis by measuring only the last millimeter of the vessel before the bifurcation. The data of the present study suggest that atherosclerosis is much more heterogeneous throughout the CCA and thus evaluation of a single site does probably not reflect the situation of the vessel at large. It appears that heterogeneity of the atherosclerotic process demands a site specific evaluation of the CCA either by serial histology or more efficient and accurate by in vivo micro-CT.

## Conclusion

Contrast enhanced micro-CT imaging enables the assessment of the vasculature and vascular complications non-invasively and in vivo. Our present study demonstrates that in vivo micro-CT is equally suited to histology for the determination of the vessel lumen.

## Disclosure

Felix Gremse is founder and owner of Gremse-IT, a startup company that offers software and services for medical image analysis in cooperation with Philips Research and the Department for Experimental Molecular Imaging.

## Supporting Information

S1 Table Regional lumen and plaque area profile of in vivo micro-CT and histology data 14 days after ligation. P-values are given for selected sections starting near the aortic arch (1), the bifurcation (9) and each 500 μm in between.
(DOCX)
