# Supplementary material for: Micro-CT Technique Is Well Suited for Documentation of Remodeling Processes in Murine Carotid Arteries
Source: PLoS One. 2015 Jun 18;10(6):e0130374. doi: 10.1371/journal.pone.0130374 (PMC4472757; doi:10.1371/journal.pone.0130374)
Supplement: S1 Table — P-values are given for selected sections starting near the aortic arch (1), the bifurcation (9) and each 500 μm in between. (DOCX) [file pone.0130374.s001.docx]

**Table S1. Regional lumen and plaque area profile of in vivo micro-CT and histology data 14 days after ligation.**

P-values are given for selected sections starting near the aortic arch (1), the bifurcation (9) and each 500 µm in between.

Day 14, lumen area, micro-CT, LCCA

| Section | 1 | 2 | 3 | 4 | 5 | 6 | 7 | 8 | 9 |
| --- | --- | --- | --- | --- | --- | --- | --- | --- | --- |
| 1 |  | 0.67 | 0.48 | 0.37 | **0.04** | 0.15 | 0.12 | 0.34 | 0.67 |
| 2 | 0.67 |  | 0.59 | 0.18 | **0.01** | 0.13 | 0.12 | 0.35 | 0.92 |
| 3 | 0.48 | 0.59 |  | **0.00** | **0.00** | **0.03** | **0.04** | 0.18 | 0.86 |
| 4 | 0.37 | 0.18 | **0.00** |  | 0.37 | 0.94 | 0.78 | 0.79 | 0.29 |
| 5 | **0.04** | **0.01** | **0.00** | 0.37 |  | 0.21 | 0.57 | 0.18 | 0.05 |
| 6 | 0.15 | 0.13 | **0.03** | 0.94 | 0.21 |  | 0.42 | 0.70 | 0.17 |
| 7 | 0.12 | 0.12 | **0.04** | 0.78 | 0.57 | 0.42 |  | 0.36 | 0.08 |
| 8 | 0.34 | 0.35 | 0.18 | 0.79 | 0.18 | 0.70 | 0.36 |  | **0.02** |
| 9 | 0.67 | 0.92 | 0.86 | 0.29 | 0.05 | 0.17 | 0.08 | **0.02** |  |

Day 14, lumen area, micro-CT, RCCA

| Section | 1 | 2 | 3 | 4 | 5 | 6 | 7 | 8 | 9 |
| --- | --- | --- | --- | --- | --- | --- | --- | --- | --- |
| 1 |  | **0.02** | **0.00** | **0.00** | **0.00** | **0.00** | **0.00** | **0.00** | **0.00** |
| 2 | **0.02** |  | **0.03** | **0.02** | **0.00** | **0.00** | **0.00** | **0.02** | **0.02** |
| 3 | **0.00** | **0.03** |  | 0.14 | 0.08 | **0.02** | 0.06 | 0.79 | 0.19 |
| 4 | **0.00** | **0.02** | 0.14 |  | 0.28 | 0.22 | 0.27 | 0.26 | 0.88 |
| 5 | **0.00** | **0.00** | 0.08 | 0.28 |  | 0.60 | 0.64 | 0.05 | 0.31 |
| 6 | **0.00** | **0.00** | **0.02** | 0.22 | 0.60 |  | 0.98 | **0.01** | 0.13 |
| 7 | **0.00** | **0.00** | 0.06 | 0.27 | 0.64 | 0.98 |  | **0.04** | 0.33 |
| 8 | **0.00** | **0.02** | 0.79 | 0.26 | 0.05 | **0.01** | **0.04** |  | 0.05 |
| 9 | **0.00** | **0.02** | 0.19 | 0.88 | 0.31 | 0.13 | 0.33 | 0.05 |  |

Day 14, lumen area, histology, LCCA

| Section | 1 | 2 | 3 | 4 | 5 | 6 | 7 | 8 | 9 |
| --- | --- | --- | --- | --- | --- | --- | --- | --- | --- |
| 1 |  | 0.84 | **0.02** | **0.02** | 0.11 | **0.01** | **0.02** | 0.99 | 0.65 |
| 2 | 0.84 |  | **0.02** | 0.09 | 0.19 | **0.01** | **0.00** | 0.91 | 0.67 |
| 3 | **0.02** | **0.02** |  | 0.53 | 0.84 | 0.30 | 0.49 | 0.19 | 0.08 |
| 4 | **0.02** | 0.09 | 0.53 |  | 0.07 | 0.97 | 0.94 | 0.11 | 0.06 |
| 5 | 0.11 | 0.19 | 0.84 | 0.07 |  | 0.35 | 0.57 | 0.14 | 0.07 |
| 6 | **0.01** | **0.01** | 0.30 | 0.97 | 0.35 |  | 0.88 | 0.09 | **0.04** |
| 7 | **0.02** | **0.00** | 0.49 | 0.94 | 0.57 | 0.88 |  | 0.13 | 0.05 |
| 8 | 0.99 | 0.91 | 0.19 | 0.11 | 0.14 | 0.09 | 0.13 |  | **0.04** |
| 9 | 0.65 | 0.67 | 0.08 | 0.06 | 0.07 | **0.04** | 0.05 | **0.04** |  |

Day 14, lumen area, histology, RCCA

| Section | 1 | 2 | 3 | 4 | 5 | 6 | 7 | 8 | 9 |
| --- | --- | --- | --- | --- | --- | --- | --- | --- | --- |
| 1 |  | **0.01** | **0.00** | **0.00** | **0.00** | **0.00** | **0.00** | **0.00** | **0.01** |
| 2 | **0.01** |  | 0.06 | **0.03** | **0.02** | **0.04** | 0.08 | 0.08 | 0.14 |
| 3 | **0.00** | 0.06 |  | 0.07 | 0.11 | 0.34 | 0.59 | 0.57 | 0.84 |
| 4 | **0.00** | **0.03** | 0.07 |  | 0.86 | 0.73 | 0.27 | 0.21 | 0.09 |
| 5 | **0.00** | **0.02** | 0.11 | 0.86 |  | 0.63 | 0.36 | 0.36 | 0.12 |
| 6 | **0.00** | **0.04** | 0.34 | 0.73 | 0.63 |  | 0.39 | 0.42 | 0.12 |
| 7 | **0.00** | 0.08 | 0.59 | 0.27 | 0.36 | 0.39 |  | 0.97 | **0.04** |
| 8 | **0.00** | 0.08 | 0.57 | 0.21 | 0.36 | 0.42 | 0.97 |  | 0.08 |
| 9 | **0.01** | 0.14 | 0.84 | 0.09 | 0.12 | 0.12 | **0.04** | 0.08 |  |

Day 14, plaque area, LCCA

| Section | 1 | 2 | 3 | 4 | 5 | 6 | 7 | 8 | 9 |
| --- | --- | --- | --- | --- | --- | --- | --- | --- | --- |
| 1 |  | 0.22 | 0.41 | 0.38 | 0.21 | 0.42 | 0.81 | 0.53 | 0.29 |
| 2 | 0.22 |  | 0.09 | 0.16 | 0.26 | 0.79 | 0.17 | 0.05 | 0.72 |
| 3 | 0.41 | 0.09 |  | 0.72 | 0.09 | 0.14 | 0.14 | 0.15 | 0.12 |
| 4 | 0.38 | 0.16 | 0.72 |  | 0.16 | 0.29 | 0.49 | 0.32 | 0.22 |
| 5 | 0.21 | 0.26 | 0.09 | 0.16 |  | 0.18 | 0.09 | **0.02** | 0.29 |
| 6 | 0.42 | 0.79 | 0.14 | 0.29 | 0.18 |  | 0.21 | 0.33 | 0.44 |
| 7 | 0.81 | 0.17 | 0.14 | 0.49 | 0.09 | 0.21 |  | 0.46 | 0.16 |
| 8 | 0.53 | 0.05 | 0.15 | 0.32 | **0.02** | 0.33 | 0.46 |  | 0.11 |
| 9 | 0.29 | 0.72 | 0.12 | 0.22 | 0.29 | 0.44 | 0.16 | 0.11 |  |
